# Patterned transparent electrode with a continuous distribution of silver nanowires produced by an etching-free patterning method

**DOI:** 10.1038/srep40087

**Published:** 2017-02-13

**Authors:** Kwonwoo Shin, Ji Sun Park, Jong Hun Han, Yunsu Choi, Dae Sung Chung, Se Hyun Kim

**Affiliations:** 1 Nano Materials & Components Research Center, Korea Electronics Technology Institute (KETI), Seongsam-si, Gyeonggi-do, 13509, Republic of Korea; 2 School of Chemical Engineering, Chonnam National University, Gwangju, 500-757, Republic of Korea; 3 School of Chemical Engineering and Material Science, Chung-Ang University, Seoul, 156-756, Republic of Korea; 4 School of Chemical Engineering, Yeungnam University, Gyeongsan, 712-749, Republic of Korea

## Abstract

The outstanding electrical, optical, and mechanical properties of silver nanowire transparent electrodes are attractive for use in many optoelectronic devices, and the recent developments related to these electrodes have led to their commercialization. To more fully utilize the advantages of this technology, developing new process technologies in addition to performance improvements is important. In this report, we propose a novel ultra-simple patterning technology to generate a silver nanowire transparent layer and a unique patterned structure with continuously distributed silver nanowires without any etched areas. The patterning is conducted by exposure to ultraviolet light and rinsing. The exposed and unexposed regions of the resulting layer have dramatically different electrical conductivities, which produces an electrical pathway without using any etching or lift-off processes. The unique patterned structure produced by this etching-free method creates hardly any optical difference between the two regions and results in excellent visibility of the patterned transparent electrode layer.

To date, indium-tin oxide (ITO), which is formed by vacuum deposition, has been primarily employed as a transparent conductive film (TCF) in electronic devices. However, the growing demands for flexible optoelectronics and lower production costs have led to research on non-ITO TCF materials^1^, such as silver nanowires (AgNWs)^2^,^3^,^4^, metal meshes^5^,^6^, carbon nanotubes^7^, conducting polymers^8^,^9^,^10^, and graphenes^11^. Among these materials, AgNW TCF technology has been successfully developed and commercialized for use in the components of touch screen panels (TSPs) on notebook PCs, tablet PCs, and smart electronics^12^,^13^.

The choice of TCF used in optoelectronic devices largely depends on its electrical conductivity and optical transparency. Generally, the TCF should have a sheet resistance of less than 200 Ω·sq^−1^ and an optical transparency of over 90%^14^. The AgNW TCF has superior optoelectronic properties: a sheet resistance of less than 50 Ω·sq^−1^ and an optical transparency of over 90%. In addition, the excellent flexibility of the AgNW TCF enables its possible application to many future flexible electronics.

For many applications, TCFs must be patterned to constitute electrical circuits. Generally, TCFs are patterned via photolithography and wet etching, which generally comprise five stages: photoresist (PR) coating on the TCF, ultraviolet (UV) light exposure, PR development, TCF etching, and PR removal^15^,^16^. Although this approach has already been established for mass production in industry, major drawbacks must be addressed: the multiple costly manufacturing steps, the use of toxic etchants, and the pattern visibility caused by the difference in reflectance between etched and non-etched regions depending on their electrode structures. Laser scribing is another method for AgNW TCF patterning. By scanning a focused nanosecond pulsed laser over the AgNW network, AgNWs are selectively ablated and patterned without requiring a conventional chemical etching or lithography step^17^. However, low productivity, which is caused by spot etching, reduces the feasibility of using this method with commercial products.

The pattern visibility problem in metallic TCFs is a major issue, especially for AgNW TCFs. Several methods have been tested to solve this problem. Regarding the wet-etching pattern, the visibility problem can be alleviated by partial etching. The partially etched nonconductive region provides a small difference in transmittance between the conductive and nonconductive regions of the TCF patterns^18^. Additionally, in laser patterning, the laser disconnects the AgNW networks in selective areas, thus electrically insulating the irradiated areas. In addition, the remaining laser-cut AgNWs reduce the optical difference between the conductive and nonconductive regions^19^. However, these methods do not fully eliminate the shortage failures between electrodes. Additionally, etching with a high-energy laser damages the plastic film substrate, which is another source of the pattern visibility problem.

Thus, in novel approaches for non-ITO TCFs, TCFs that possess effective processability in patterning and excellent clear visibility are highly desired. Here, we demonstrate a novel TCF technique using a photosensitive AgNW composite layer.

The photosensitive AgNW composite layer can be coated using a simple solution process, such as bar coating, slot-die coating, micro-gravure, spray coating, or spin coating, with a photosensitive AgNW composite solution. The coated composite layer is sensitive to UV; as a result, the film can be patterned by UV exposure. When a specific region of the photosensitive AgNW composite layer is exposed to UV light, the photopolymers in the exposed region are cross-linked and produce nonconductive regions. The composite film contains exposed and unexposed regions with dramatically different electrical properties. The exposed region exhibited insulating characteristics and a sheet resistance of over 20 MΩ·sq^−1^, which was the limit of the measurement instrument. The unexposed region exhibited low resistance, i.e., less than 50 Ω·sq^−1^, which produced an electrical pathway for AgNW transparent electrodes following a very simple exposure process.

In a patterned TCF, the AgNWs are not removed, broken or etched and are distributed uniformly across the whole surface in both the conducting and insulating regions. This unique structure hardly creates an optical difference between each region, unlike the common patterning techniques that use etching methods, which provide very transparent and clear visibility without any recognition of the pattern electrodes.

## Results and Discussion

**Figure 1:**
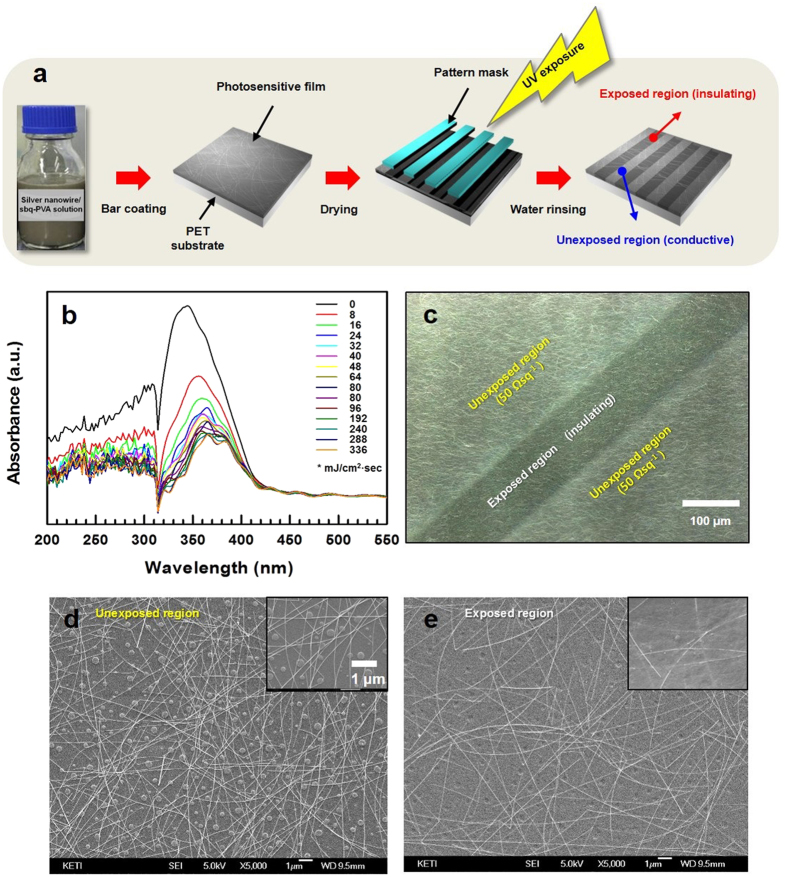
(**a**) The preparation procedure for the patterned transparent electrode layer with a continuous distribution of AgNWs. (**b**) The dependence of the UV absorbance on the exposure dose. (**c**) The confocal microscopy image of the patterned layer. (**d**,**e**) The SEM images of the unexposed and exposed regions in the patterned layer, respectively. The inset of each image is a magnification.

The procedure for patterning the photosensitive AgNW composite layer is shown in Fig. 1(a). The composite coating solution was prepared by combining AgNW, hydroxypropyl methylcellulose (HPMC), polyethylenimine (PEI), ethylene glycol, and poly(vinyl alcohol), N-methyl-4(4′-formylstyryl) pyridinium methosulfate acetal (sbq-PVA). The prepared coating solution was homogeneously coated on polyethylene terephthalate (PET) substrates via the bar-coating method. The coated substrate was baked at 110 °C in a convection oven for 10 minutes. Finally, the composite film was exposed to UV light and rinsed with water (see Video File 1 in the supporting information).

In the coating solution, AgNWs are present as electrically conductive nanoparticles that make up a conductive surface layer on the substrate. HPMC is a viscosity modifier that enables the coating of various substrates and enhances wettability. PEI is a polyelectrolyte material, and its sufficient adsorption characteristic facilitates good adhesion of the AgNWs to the substrate. Ethylene glycol improves the leveling of the coated wet surface and enhances the uniformity of the resistance. sbq-PVA is a water-soluble photosensitive polymer that can be cross-linked by UV light (300–420 nm).

In the coated composite layer, the photosensitive sbq-PVA polymers absorb UV light and simultaneously form cross-links. Previous research has studied the photocrosslinking behavior of sbq-PVA. According to Nam *et al*.^20^, the reaction of the C=C double bonds in the 4(4′-formylstyryl) pyridinium methosulfate salts forms the [2 + 2]-cycloaddition products and crosslinks the polymer chains. To identify the optimum exposure dose for the cross-linking of the photosensitive polymers, the dependence of the light absorbance on the UV exposure dose was investigated, as shown in Fig. 1(b). The peak between 310–400 nm decreased with the incident UV exposure dose, and the absorbance was almost saturated at an exposure dose of approximately 24 mJ cm^−2^.

To create a specific pattern, a specific region of the photosensitive composite layer was exposed to UV light using a pattern mask. Then, the composite layer was rinsed with distilled water to terminate the UV reaction. During rinsing, unreacted sbq-PVA in the unexposed region was removed, and the UV reaction was terminated. The resulting layer had a patterned structure, as shown in Fig. 1(c). The unexposed region exhibited conducting properties with a sheet resistance of 50 Ω·sq^−1^, and the exposed line region was nonconductive, with a sheet resistance of over 20 M Ω·sq^−1^. In Fig. 1(c), the exposed region cut an electric pathway between two conducting (unexposed) regions. In the unexposed region, unreacted sbq-PVA was removed during rinsing, and an electrically connected AgNW network layer was produced, as shown in Fig. 1(d). However, in the exposed regions, sbq-PVA was cross-linked by UV light and was not removed during rinsing. AgNWs exist in the middle of the polymeric matrix of the cross-linked sbq-PVA, and the interconnections among AgNWs are disturbed by the existing polymeric matrix, which produces electrically insulating regions, as shown in Fig. 1(e). The particulate substance in Fig. 1(d) is HPMC, which acts as a viscosity modifier in the coating solution. While baking the coated composite layer, the HPMC and sbq-PVA were phase-separated, which produced HPMC with a particulate shape. Because HPMC dissolves very slowly in water, it is not removed during rinsing. Although the particulate substances can increase the haze of a transparent layer, the over-coating in the subsequent step can diminish this effect on the optical properties. In fact, the haze decreased from 1.2% to 1% after over-coating.

**Figure 2:**
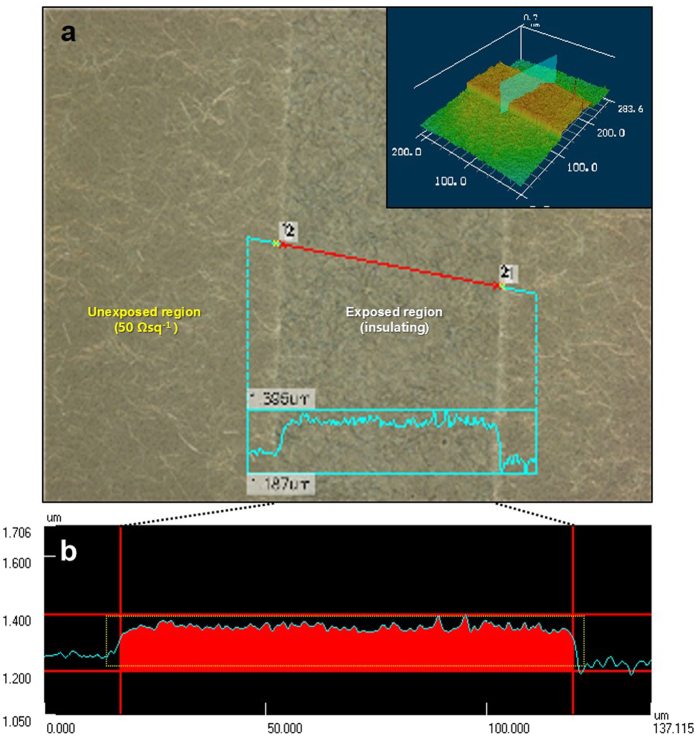
(**a**) The confocal optical microscopy images of the patterned transparent electrode layer with a continuous distribution of AgNWs. The inset is the 3D confocal image. (**b**) The cross-sectional image of the exposed region of (**a**).

The difference in the solubility of sbq-PVA between the regions was confirmed by the different thicknesses. As shown in Fig. 2, which presents a confocal optical microscopy image and its cross-sections, the exposed region was thicker than the unexposed region, which created a step (height of 0.1–0.15 μm) at the boundary (Fig. 2(b)). Because of the cross-linking due to UV exposure, the photosensitive polymers in the exposed region were not removed during rinsing and produced a thicker insulating region. The cross-section shows a reverse thickness profile compared to the etched pattern. In the etched pattern, the insulating region, which was the etched region, either did not have a thickness or was a very thin layer, and the conductive electrode region had greater thickness.

**Figure 3:**
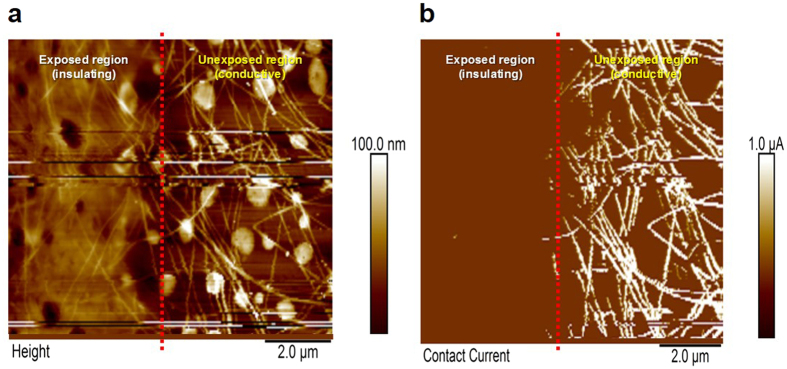
The AFM images of the boundary between the exposed and unexposed regions. (**a**) The image captured in the height mode. (**b**) The image captured in the current mode. The white line in (**b**) shows the electrical current pathways.

Figure 3 displays the atomic force microscopy (AFM) topographies (height mode, conducting mode) of the boundary. As shown in the height-mode topographic image, the AgNWs were located all over the surface and boundary. However, in conducting mode, the current mapping of each region revealed a different electrical connectivity in each region. The unexposed region had a high current density, in accordance with the distributed AgNWs. However, there was no electrical conductivity in the exposed region, and the AgNWs themselves did not have a remarkable current density because they were nearly all buried in the middle of the cross-linked sbq-PVA. The applied voltage difference between the AFM tip and substrate was 4 V in current mode.

In the boundary between the exposed and unexposed regions, an intermediate region approximately 1 μm wide was observed, as indicated by the red dotted line in Fig. 3. The intermediate region had transient conductivity, as shown by the current density in that region. Although the presence of an intermediate region can restrict a more precise pattern, most applications, such as TCFs, displays, solar cells, and organic light emitting diodes (OLEDs), are not affected by the intermediate region. In the application of TSPs in modern high-end smart phones, the electrode width and space are over 100 μm and 30 μm, respectively. Other applications generally require patterned electrodes on the scale of a few hundreds of micrometers or millimeters.

In the patterned film, the minimum space between conducting regions, or the minimum width of the insulating region, was largely dependent on the length and density of the AgNWs. The isolated electrode patterned by our etching-free method can be directly electrically connected by abnormally long AgNWs. Therefore, the maximum length of the AgNWs determines the minimum width of the space. Although uniform AgNW materials can be applied, the abnormally long AgNWs must be removed or broken. Ultrasonic treatment can reduce the number of long AgNWs. As shown in Figure S1, AgNWs were broken as the ultrasonic treatment time increased. However, too much treatment increased the sheet resistance of the TCF. At 30 seconds of treatment, the sheet resistance was over 400 Ω·sq^−1^. An ultrasonic was applied at a power of 400 W. Without the ultrasonication, our patterning method could provide well-controlled electrical connectivity over a 100 μm pattern space. However, there were a few shortage failures in a narrow pattern space of 70 μm. With ultrasonication for 10 seconds, there were no shortage failures in the 70 μm pattern space. Therefore, an optimal length of 10–20 μm, which is the provided specification for AgNW products, is suggested for a pattern space of over 100 μm and a sheet resistance of 40–50 Ω·sq^−1^.

**Figure 4:**
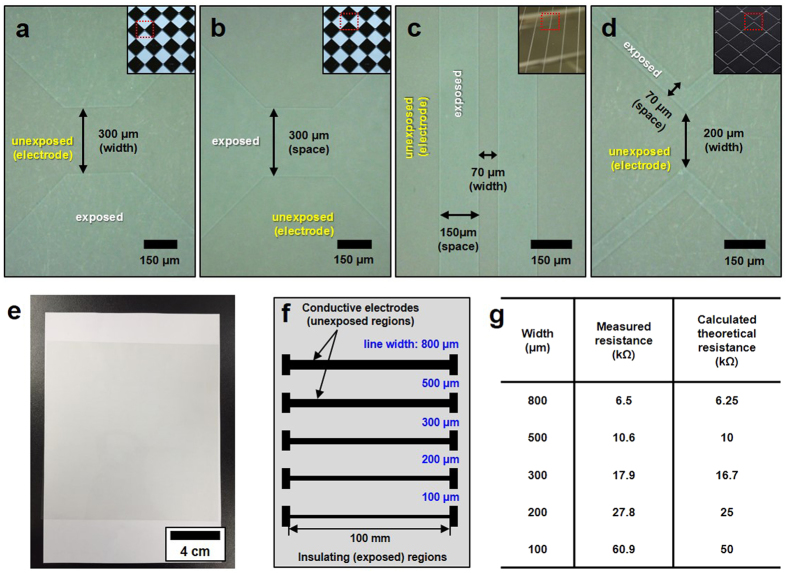
(**a**–**d**) The optical microscopy images of the patterned layer. The inset of each image is a picture of the mask pattern. (**e**) The picture of the patterned transparent electrode film with dimensions of 200 mm*200 mm. (**f**) The test patterns of bar-type electrodes with different electrode widths. (**g**) The measured electrical resistance and the calculated resistance of each bar-type electrode with different widths shown in (**f**).

Diverse electrode shapes, such as diamond and bar patterns, were introduced on the TCF. The pattern structures were investigated by confocal optical microscopy. Because of the difference in polymer distribution between the exposed and unexposed regions, the brightness contrast between their surfaces was clearly distinguishable. Figure 4(a) and (b) show diamond-patterned electrodes with a 300 μm minimum line width and space, respectively. Line-patterned electrodes with a 70 μm line width and a 150 μm line space are presented in Fig. 4(c), and those with a 200 μm minimum line width and 70 μm line space are shown in Fig. 4(d). A photograph of the patterned AgNW composite film on the PET substrate (200 mm × 200 mm) is shown in Fig. 4(e). The measured transmittance and haze of the TCF (including the PET substrate) were 90.5% and 1.2%, respectively. The bare PET before application of the AgNW composite coating had 92.1% transmittance and 0.41% haze.

**Figure 5:**
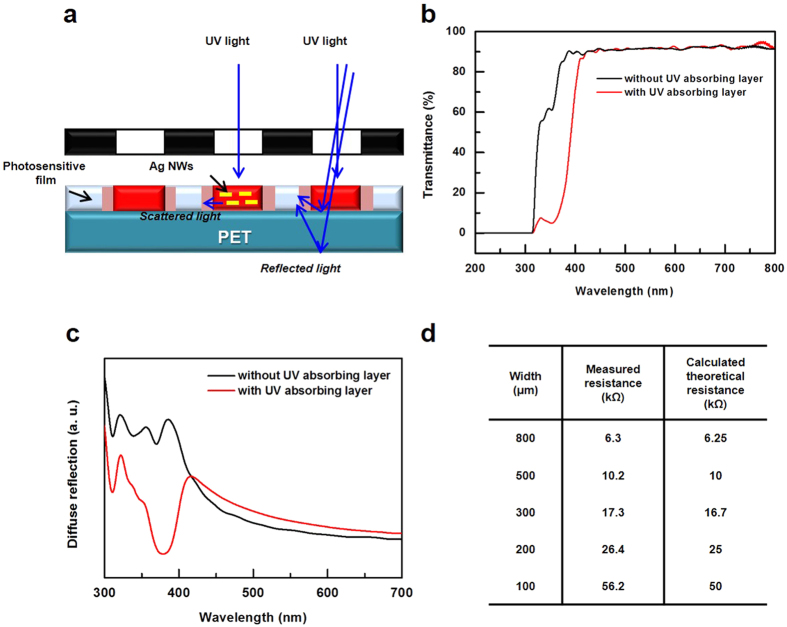
(**a**) An illustration showing that the UV light scattered and reflected by the AgNWs and the interfaces during the exposure step. (**b**) The transmittance spectra of the PET substrates. The black trace is without the UV-absorbing layer, and the red trace is with the UV-absorbing layer. (**c**) The diffuse reflection spectra of photosensitive the AgNW composite layers on each substrate. (**d**) The measured electrical resistance and the calculated resistance of each bar-type electrode with different widths shown in Fig. 5(f).

In our experiment, the exposure dose for fine patterns was optimal in the range of 10–15 mJ cm^−2^. A dose exceeding this range can disturb the formation of fine patterns. The light that was scattered and reflected by the AgNWs in the composite layer and the interface between the composite layer and substrate disturbed the delicate exposure to UV light near the pattern edge, as illustrated in Fig. 5(a). Therefore, minimized exposure is better for a fine pattern, if sbq-PVA is cross-linked and not removed during rinsing. Additionally, another UV exposure can be applied to the patterned film after rinsing to confirm the reaction of sbq-PVA.

To confirm the electrical resistance of the prepared electrodes, several channels with various line widths (100–800 μm) were formed on the TCF with a sheet resistance of 50 Ω·sq^−1^, as shown in Fig. 4(f). The measured line resistance matched the calculated values well, as shown in Fig. 4(g). However, at a narrow width (100 μm, 200 μm), the measured resistance was relatively higher than the calculated values. This difference originated from the UV light that was scattered and reflected by the AgNWs and interface, which disturbed the delicate exposure to UV light near the pattern edge and hindered the formation of clear boundaries. As a result, the effective electrode width decreased, and the electrode resistance increased. This phenomenon has a greater effect on relatively narrow electrodes.

For far more delicate patterns, the introduction of a UV-absorbing layer between the photosensitive composite layer and substrate could enhance the degree of pattern precision. The UV-absorbing layer absorbs the scattered and reflected UV light, which enables more precise exposure near the pattern boundary. In our experiment, a commercially available UV-absorbing solution (HC600) was spin-coated on a PET substrate and cured at 130 °C in an oven for 5 minutes. The PET substrate with a UV-absorbing layer showed low transmittance in the wavelength range of 300–420 nm, as shown in Fig. 5(b). To observe the relative amounts of scattered and reflected UV light depending on the UV-absorbing layer, the diffuse reflectance was measured. The photosensitive AgNW composite film was coated on each substrate, and the diffuse reflection was analyzed depending on the presence of the UV-absorbing layer, as shown in Fig. 5(c). In a composite layer with the UV-absorbing layer, the peak corresponding to the absorption of the diffuse reflection was observed in the wavelength range of 300–420 nm, indicating that the UV-absorbing layer effectively absorbed the UV light that was scattered and reflected by the AgNWs and interfaces and decreased the diffuse reflection in the wavelength range of 300–420 nm.

To identify the effect of the UV-absorbing layer on electrode resistance, several electrodes were prepared with various line widths (100–800 μm), as shown in Fig. 4(f), and the electrical resistances of these electrodes are compared in Fig. 5(d). The measured values matched better than the calculated values. Additionally, the resistance of the pattern with a UV-absorbing layer shown in Fig. 5(d) was closer to the calculated resistance than that of the pattern without a UV-absorbing layer shown in Fig. 4(f).

To improve the optical and electromechanical properties, the patterned layer was overcoated with polyurethane. The overcoating was performed by spin-coating a polyurethane solution (1 wt%) and drying for 5 minutes in a convection oven at 130 °C. After overcoating, the patterned film became clearer, with an increase in transparency to 91% and a decrease in haze to 1.0. The overcoated patterned film provided clear visibility, and the patterns were nearly invisible. The surface resistance was maintained at 50 Ω sq^−1^ because the top coating was very thin, i.e., approximately 50 nm.

**Figure 6:**
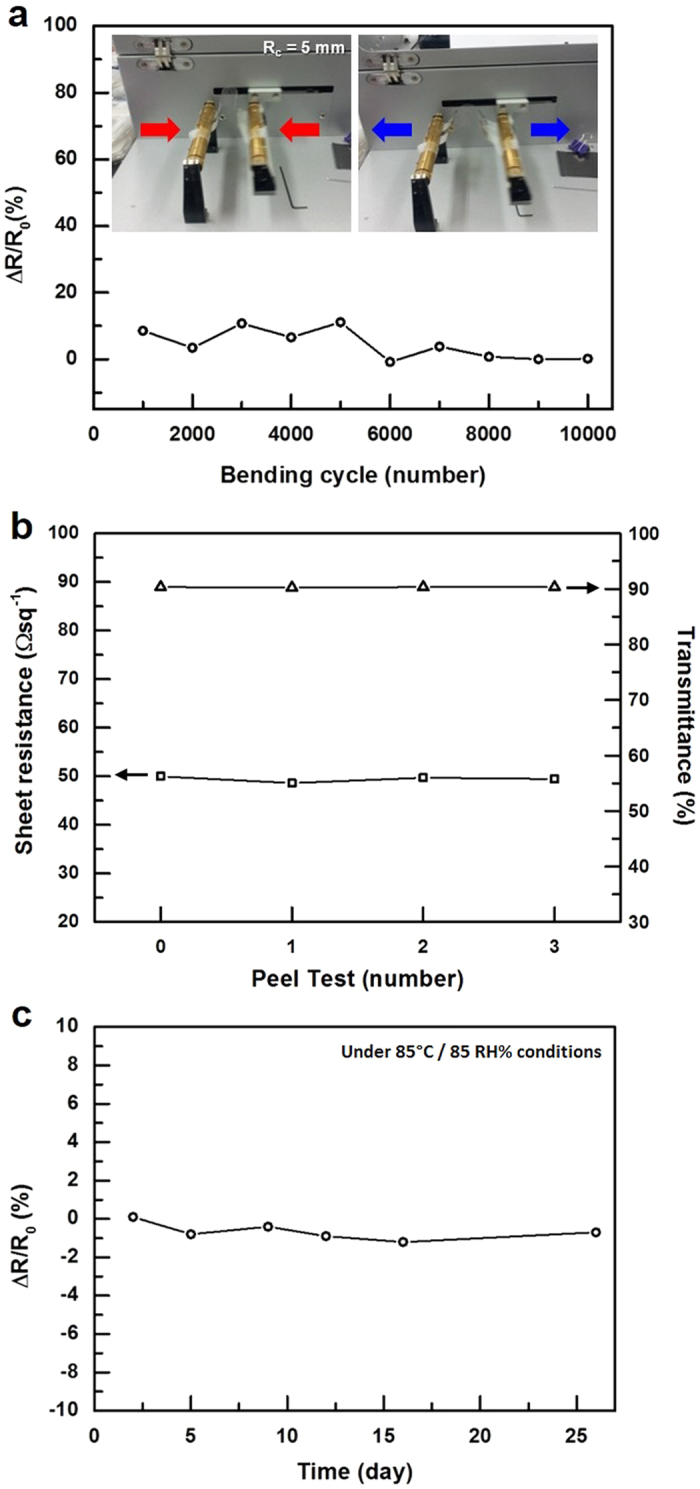
The electromechanical properties of the transparent AgNW electrode. (**a**) The resistance variations depending on the number of bending deformations. The bending radius was 5 mm. (**b**) The resistance and transmittance variations depending on the number of peel tests. (**c**) The resistance variations during the long-term environmental stability test under high temperature and high humidity conditions (85 °C, 85% relative humidity).

The electromechanical properties of our AgNW transparent film are presented in Fig. 6 (see the detailed test conditions and explanations in the supporting information).

Although the patterns could be discerned from very careful observation because of the steps in the boundary and different surface topographies in each region, the patterned layer provided very clear visibility without any recognition of the patterns after attachment to a 30-μm-thick optically clear adhesive (OCA) film, regardless of their sizes. The attached OCA film diminished the height of the steps at the boundary and provided a similar surface morphology in both regions. In contrast, for the ordinary AgNW pattern created by the etching process could easily be observed with the naked eye. In addition, the OCA film did not diminish the difference in optical properties between the etched and un-etched regions. According to our experiment, the etched pattern was recognizable with a pattern space of over 30 μm.

**Figure 7:**
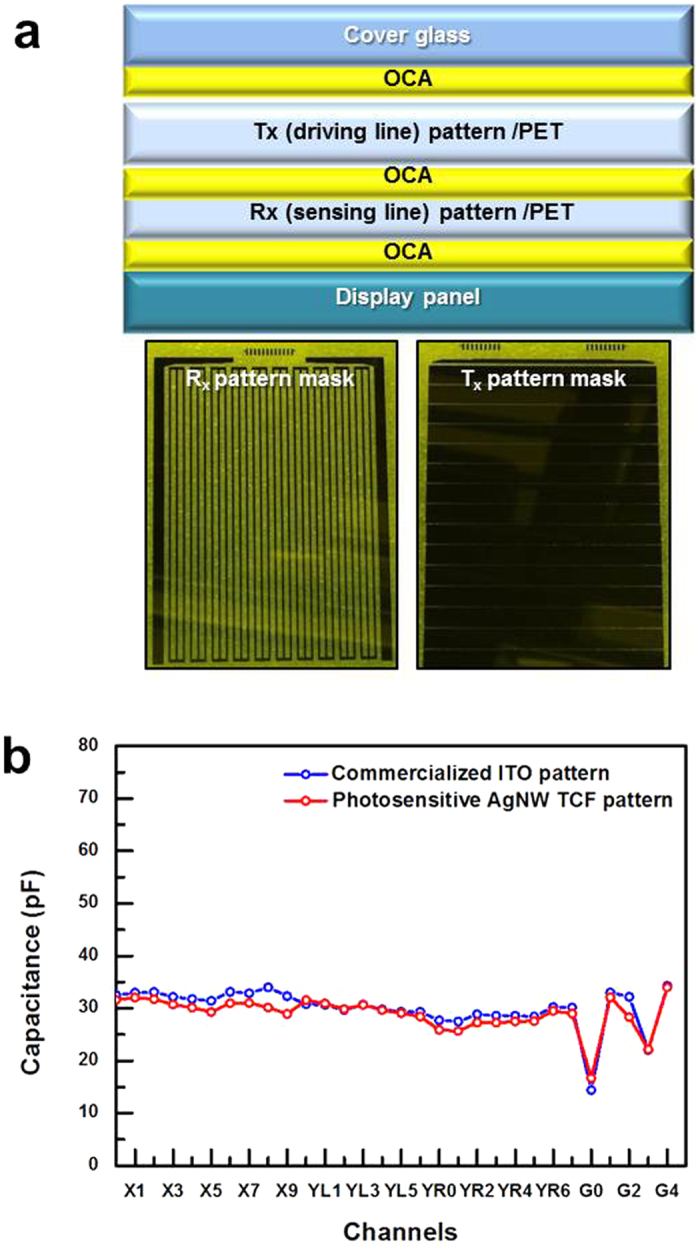
(**a**) The upper figure shows the structure of the GFF-type TSP, which consisted of a cover glass and two TCF films. The lower figures show the R_x_ pattern (sensing line, *y*-axis pattern) and T_x_ pattern (driving line, *x*-axis pattern). (**b**) The capacitance of each electrode node of the TSP sensor, and a comparison of our TSP with the commercialized ITO one.

The TSP of a modern smart phone was made using our photosensitive AgNW TCF and etching-free patterning method. The structure of the TSP was the GFF type, which consists of a cover glass and two TCF films. The TCF films provided an R_x_ pattern (sensing line, *y*-axis pattern) and a T_x_ pattern (driving line, *x*-axis pattern), as shown in Fig. 7(a). The TSP sensor was prepared by coupling the R_x_-patterned film and T_x_-patterned film with a 30-μm-thick OCA film. The TCF sensor was analyzed by measuring the capacitance of each electrode node, as shown in Fig. 7(b). To confirm the properties of the TSP sensors, our TSP sensor was compared with a commercialized ITO TSP sensor in the same device, and the capacitance responses of each node were very similar to each other. This result indicates that our photosensitive film and novel patterning method can be used to attain an electrical pathway in commercial TSP products. The smart phone was operated normally with our TSP sensor at the level of a commercialized product. The visibility was excellent without any recognition of the patterns, and the transmittance of the overall TSP modules was 87.2%.

We developed a novel etching-free patterning method to create an AgNW transparent layer. We produced a patterned layer with a unique structure and demonstrated its use in real applications. The method is very simple, effective, and environmentally friendly because it does not use any toxic etchants and can also be applied in lower-resistance applications, such as OLED lighting, displays and organic photovoltaic devices. We used the transparent electrode in OLED lighting with a 20 Ω/sq-sheet-resistance film and produced a successful patterning result. Currently, we are working to apply the electrode in an organic photovoltaic device a with 15 Ω/sq-sheet-resistance film.

Nevertheless, the precision of the patterns is still not sufficient for other applications. The patterns could be improved by using shorter AgNWs with smaller diameters. The short lengths should reduce the electric shortage failures in narrow pattern spaces, which would improve the precision of the patterning. AgNWs with smaller diameters can also provide superior optical properties. Thus, the negative effects on conductivity due to short AgNWs can be compensated by applying more AgNWs with smaller diameters.

## Materials

AgNWs (lengths of 10–20 μm and diameters of 20 nm) were purchased from Aiden (Korea). Poly(vinyl alcohol),N-methyl-4(4′-formylstyryl) pyridinium methosulfate acetal (M_w_ approximately 45,000) was purchased from Polyscience, Inc. (USA). Other chemicals were purchased from Sigma-Aldrich (USA). HC600 was purchased from JTS KOREA (Korea).

### Preparation of photosensitive silver nanowire composite solution

First, 0.8 g of an aqueous AgNW dispersion (0.5 wt%) was mixed with 1 g of hydroxypropyl methylcellulose solution (0.175 wt%). Then, 0.4 g of sbQ-PVA aqueous solution (4.4 wt%) and 0.4 g of polyethylenimine aqueous solution (0.25%) were added. Finally, 0.26 g of ethylene glycol was subsequently added. After the preparation of the solution, it was homogeneously blended by mild shaking.

### Coating/Patterning of photosensitive AgNW composite film

1. Coating: A photosensitive composite film was coated on a polyethylene terephthalate (PET) substrate through a bar-coating method, and then it was dried for 10 minutes in a 110 °C oven.
2. Patterning: To pattern the composite film, a pattern mask was placed on the film and exposed to parallel UV light (365 nm wavelength). The exposed film was rinsed with water for 10 seconds and dried under a stream of air.

### Characterization

Sheet resistance was measured using a four-point probe system (MCP-T360, Mitsubishi Chemical). Light absorbance and diffuse reflectance were measured using a UV-VIS spectrometer (Jasco, V530). The transmittance and haze of the TCF were measured using a Haze meter (NDH-5000, NIPPON DENSHOKU INDUSTRIES Co., LTD.). The morphologies of the patterned electrodes were investigated using a field emission scanning electron microscope (FE-SEM, Hitachi S-4800, accelerating voltage: 15 kV) and a confocal optical microscope (Zeiss, LSM320). The surface morphologies and relative conductivity values of the TCF were analyzed by atomic force microscopy (Bruker, Nanoscope V multimode 8). The environmental stability of the composite film was assessed in a temperature and humidity test chamber (Won Tech, WTH-300) for 30 days.

## Additional Information

**How to cite this article:** Shin, K. *et al*. Patterned transparent electrode with a continuous distribution of silver nanowires produced by an etching-free patterning method. *Sci. Rep.* **7**, 40087; doi: 10.1038/srep40087 (2017).

**Publisher's note:** Springer Nature remains neutral with regard to jurisdictional claims in published maps and institutional affiliations.

## Supplementary Material

Supplementary Information

Supplementary Video 1
