# Supplementary material for: Patterned transparent electrode with a continuous distribution of silver nanowires produced by an etching-free patterning method
Source: Sci Rep. 2017 Feb 13;7:40087. doi: 10.1038/srep40087 (PMC5304198; doi:10.1038/srep40087)
Supplement: Supplementary Information [file srep40087-s1.pdf]

Patterned transparent electrode with continuous distribution of silver nanowires produced by etching-free patterning method

By: *Kwonwoo Shin*, Ji Sun Park, Jong Hun Han, Yunsu Choi, Dea Sung Chung,* and *Se Hyun Kim*

W. Shin, J. S. Park, Y. Choi
Nano Materials & Components Research Center
Korea Electronics Technology Institute (KETI)
Seongsam-si, Gyeonggi-do, 13509, Republic of Korea
E-mail: kwshin@keti.re.kr

J. H. Han
School of Chemical Engineering
Chonnam National University, Gwangju
500-757, Republic of Korea

D. S. Chung
School of Chemical Engineering and Material Science
Chung-Ang University, Seoul
156-756, Republic of Korea

S. H. Kim
School of Chemical Engineering, Yeungnam University, Gyeongsan
712-749, Republic of Korea

Supplementary information

In Figure 6(a), the bending test of the transparent film was performed for over 10,000 bending cycles at a 5 mm bending radius. After each cycle, the electrical resistance of the electrode was automatically measured through the metal electrodes connected to both ends of the TCF. The average of resistance during every 1,000 cycles was plotted on the graph. Although there was a change in resistance as high as 10% from the mechanical vibrations and shocks of the connecting terminal, the resistance of our TCF was maintained within the range of 0.2% after

10,000 bending cycles. The adhesion of the TCF on a PET substrate was investigated in Figure 6(b) by means of a peel test. 3M-Scotch tape was applied on the AgNW TCF by pressing it with a finger and detaching it from the PET substrate. As a result, the sheet resistance and transmittance of TCF were maintained after the peel test. Moreover, a long-term environmental stability test of the patterned AgNW TCF was performed under high temperature/high humidity (85°C and 85% relative humidity) conditions for 28 days (Figure 6(c)). The sheet resistance was uniformly maintained in the high temperature/high humidity conditions. These results suggest that our photosensitive AgNW TCF has excellent electromechanical properties.

Figure S1

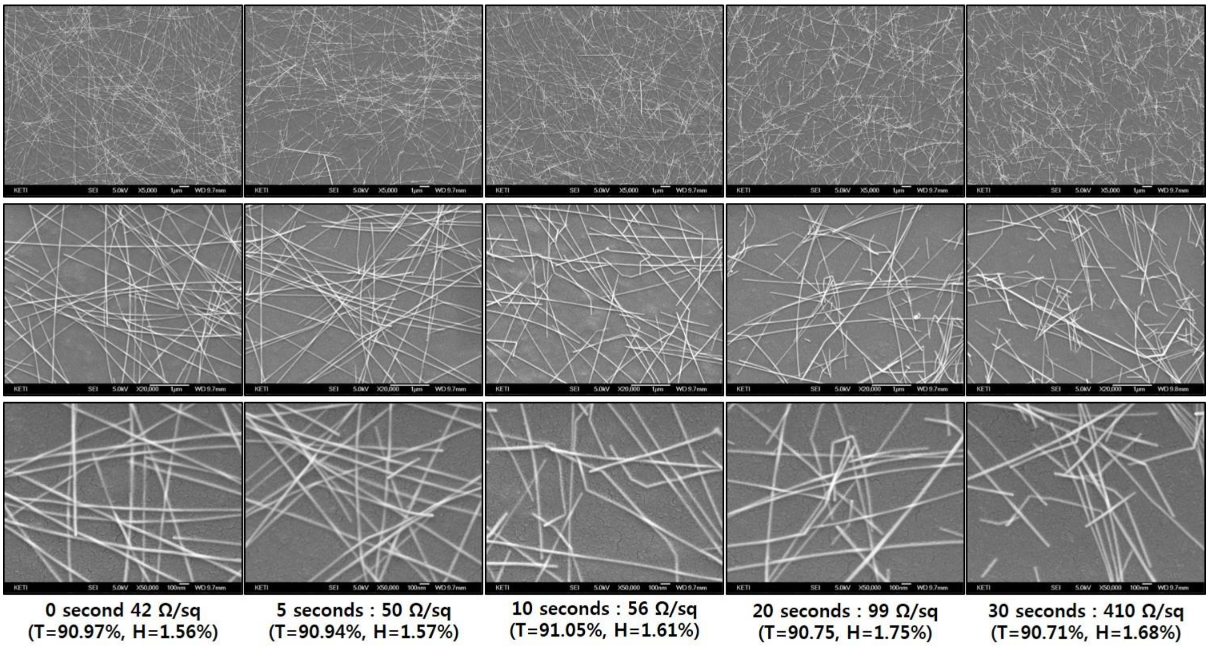

Figure S1. The SEM images of AgNWs and optoelectricc properties depending on ultrasonic treating times at power 400W. T is transmittance and, H is haze.

Supplementary video 1

The procedure of preparation of the patterned transparent electrode layer with continuous distribution of silver nanowires.

(a) Coating step: The photosensitive composite layer was coated on polycarbonate substrate with bar-coating method.

(b) Bake step: The coated substrate was was baked at 110 °C in a convection oven for 10 minutes.

(c) UV exposure step: The baked substrate was placed on pattern mask, and was exposed to UV on a specific region using a pattern mask.

(d) Termination step: The exposed substrate was dipped in water. In this step, un-reacted sbq-PVA in the unexposed region is removed and UV reaction is terminated.

(e) drying step: remove water and dry the patterned substrate.

(3) Measuring step: Measure the channel resistacne (~2 kΩ) and chech the insulating property between adjacent channels (O.L, over load; >30 MΩ).
